# Valproic acid sensitizes metformin-resistant human renal cell carcinoma cells by upregulating H3 acetylation and EMT reversal

**DOI:** 10.1186/s12885-018-4344-3

**Published:** 2018-04-17

**Authors:** Muyun Wei, Shaowei Mao, Guoliang Lu, Liang Li, Xiaopeng Lan, Zhongxian Huang, Yougen Chen, Miaoqing Zhao, Yueran Zhao, Qinghua Xia

**Affiliations:** 1 Department of Center Laboratory, Shandong Provincial Hospital Affiliated to Shandong University, 544 Jingsi Road, Jinan, 250001 Shandong Province China; 2 Minimally Invasive Urology Center, Shandong Provincial Hospital Affiliated to Shandong University, 9677 Jingshidong Road, Jinan, 250001 Shandong Province China; 3 Department of Urology, Qingdao center Hospital, Qingdao, 266042 Shandong Province China; 4 Department of Urology, Jinan center Hospital, Jinan, 250001 Shandong Province China; 5 Department of pathology, Shandong Provincial Hospital Affiliated to Shandong University, 324, Jingwu weiqi Road, Jinan, 250001 Shandong Province China

**Keywords:** Metformin, Valproic acid, Histone H3, EMT, Resistance

## Abstract

**Background:**

Metformin (Met) is a widely available diabetic drug and shows suppressed effects on renal cell carcinoma (RCC) metabolism and proliferation. Laboratory studies in RCC suggested that metformin has remarkable antitumor activities and seems to be a potential antitumor drug. But the facts that metformin may be not effective in reducing the risk of RCC in cancer clinical trials made it difficult to determine the benefits of metformin in RCC prevention and treatment. The mechanisms underlying the different conclusions between laboratory experiments and clinical analysis remains unclear. The goal of the present study was to determine whether long-term metformin use can induce resistance in RCC, whether metformin resistance could be used to explain the disaccord in laboratory and clinical studies, and whether the drug valproic acid (VPA), which inhibits histone deacetylase, exhibits synergistic cytotoxicity with metformin and can counteract the resistance of metformin in RCC.

**Methods:**

We performed CCK8, transwell, wound healing assay, flow cytometry and western blotting to detect the regulations of proliferation, migration, cell cycle and apoptosis in 786-O, ACHN and metformin resistance 786-O (786-M-R) cells treated with VPA, metformin or a combination of two drugs. We used TGF-β, SC79, LY294002, Rapamycin, protein kinase B (AKT) inhibitor to treat the 786-O or 786-M-R cells and detected the regulations in TGF-β /pSMAD3 and AMPK/AKT pathways.

**Results:**

786-M-R was refractory to metformin-induced antitumor effects on proliferation, migration, cell cycle and cell apoptosis. AMPK/AKT pathways and TGF-β/SMAD3 pathways showed low sensibilities in 786-M-R. The histone H3 acetylation diminished in the 786-M-R cells. However, the addition of VPA dramatically upregulated histone H3 acetylation, increased the sensibility of AKT and inhibited pSMAD3/SMAD4, letting the combination of VPA and metformin remarkably reappear the anti-tumour effects of metformin in 786-M-R cells.

**Conclusions:**

VPA not only exhibits synergistic cytotoxicity with metformin but also counteracts resistance to metformin in renal cell carcinoma cell. The re-sensitization to metformin induced by VPA in metformin-resistant cells may help treat renal cell carcinoma patients.

## Background

Renal cell carcinoma (RCC) is the predominant form (approximately 85%) of kidney cancer in adults [1]. Although RCC takes the third place in incidence among urologic tumors, it is the worst in cancer specific mortality, since it has a poor prognosis and more than 40% of patients with RCC die within 5 years after diagnosis, opposite to the 20% mortality observed in prostate cancer or bladder carcinoma [2]. Surgery is the primary method to treat RCC, however there still are 30%–40% of patients develop metastases or recurrence after surgery [3]. In addition, RCC shows resistance to chemotherapy and radiation treatment. Therefore, to discover novel therapeutic strategies of RCC is urgently needed.

Metformin (Met), because of relatively inexpensive, safe, and well tolerated, is recommended as the first glucose-lowering treatments and the most commonly prescribed oral antidiabetic agents for type 2 diabetes [4]. There were numerous experimental studies suggested that metformin exerts anti-tumour effects in various cancer cell lines, including the endometrium [5], bladder [6], colon [7], ovarian [8], lung [9], breast [10], stomach [11], prostate [12], as well as RCC [13–15]. But, in studies that epidemiologically and observationally analysed whether metformin use in patients could be associated with the risk of cancer, the conclusions were quiet variant. Some of these studies showed evidence of a decrease in cancer risk when using metformin [16–18], while more studies indicated that metformin therapy was not significantly associated with lower cancer risk in endometrial cancer [19], bladder cancer [20], thyroid cancer [21], lung cancer [22], and prostate, breast, and colorectal cancer [23–25]. This inconformity was also observed in RCC. Several epidemiological studies showed that the use of metformin was not significantly associated with the kidney cancer outcomes as well as the risk of death [26–31], while Tseng et al. and Li et al. found that metformin use is correlated with improved survival in patients with localized RCC, but not in metastatic RCC [32, 33]. Although studies in types of cancers and RCC lines suggested that metformin has remarkable antitumor activities, making metformin seems to be promising as a cancer chemo preventive or therapeutic drug, the fact that metformin might not be effective in reducing the risk of RCC in cancer clinical trials makes it difficult to determine the benefits of metformin in RCC prevention and treatment. The mechanisms underlying the difference between in vitro experiments and in vivo analysis remains unclear.

It is well documented that one of the key targets of metformin is adenosine monophosphate-activated protein kinase (AMPK), which inhibits the mammalian target of rapamycin (mTOR) and therefore suppresses cell proliferation, induces apoptosis and upregulates tumour suppressor genes and proteins [34]. In addition, metformin can reduce the activation of insulin pathway proteins such as protein kinase B (AKT), extracellular regulated protein kinases (ERK) and the activity of transforming growth factor β (TGF-β) induced epithelial-to-mesenchymal (EMT). Long-term administration of low-dose metformin to patients is safe, but the drug resistance response of tumour also appears. Laboratory experiments performed on metformin and RCC mainly concern the short-term impacts of metformin, but the treatments of metformin in clinical analysis are usually long-term administrations. Therefore, we hypothesis that the inconformity of metformin’s antitumor activities between in vitro experiments and in vivo analysis is caused by the metformin resistance in tumour cells.

Research suggested that valproic acid (VPA), which was used as an anticonvulsant drug for years and defined as a broad-range histone deacetylases inhibitor (HDACi), was related with inhibitions on cell proliferation and differentiation, cell cycle control, DNA repair, and apoptosis in RCC cells [35]. VPA can activate ERK and AKT proteins and perform antitumor effects by regulating both caspase-dependent and –independent apoptotic signal pathways [36, 37]. Earlier reports demonstrated that VPA and metformin combination leads cell cycle arrest and cell apoptosis in RCC cells [38]. The present study showed that long-term metformin treatment of RCC cells cause resistance to metformin. The drug resistance was accompanied by specific changes in acetyl-Histone 3 (aH3), AMPK/AKT and TGF-β/SMAD3 pathways. Results of our study showed that, comparing with one drug alone, the VPA and metformin combination strategy not only enhanced the antitumor activity but also overcame the resistance induced by the long-term use of metformin in RCC cells.

## Methods

### Cell lines and reagents

Kidney carcinoma Caki-1 (HTB-46), ACHN (CRL-1611), 786-O (CRL-1932), and A498 (HTB-44) cells were purchased from the China Center for Type Culture Collection (wuhan, China). 786-O cells were cultured in RPMI-1640 medium (Thermo Fisher Scientific, Waltham, MA, USA), ACHN and A498 in MEM medium (Thermo Fisher Scientific), Caki-1 cells in McCoy’s 5A medium(Thermo Fisher Scientific), supplemented with 10% foetal bovine serum (FBS,Thermo Fisher Scientific) at 37 °C in a humidified, 5% CO_2_ incubator. Metformin (sigma Chemical Co., St. Louis, MO, USA) was dissolved in PBS at a store concentration of 1 M. VPA (Sigma) was diluted in PBS at a store concentration of 200 mM. LY294002, Rapamycin and AKT inhibitor (CST, Cell Signaling Technology, Danvers, MA, USA), SC79 (sigma) were diluted in DMSO and store in − 80 °C. Purified recombinant human TGF-β (Sigma) was diluted in PBS at a store concentration of 50 mg/ml.

### Establishment of metformin-resistant cells

The half maximal inhibitory concentration (IC50) of RCC cells to metformin was determined by incubating cells with different concentrations of metformin in 96-well plates. 786-O, ACHN, Caki-1 and A498 cells were exposed to VPA (0.1, 0.5, 1, 5, 10, 20, 40, 60, 80 and 100 mM), metformin (1, 5, 10, 20, 40, 60, 80 and 100 mM) or phenformin (0.1, 0.2, 0.5, 1, 2, 5, 10, 20 mM). After 48 h treatment, the cell count was detected by Cell Counting Kit-8 (CCK8) (DOJINDO, Kumamoto, Japan). For establishing the metformin-resistant cells, the 786-O cells were cultured in a 25 mm^2^ bottle and incubated in medium with metformin at a concentration just below the IC50 values. The metformin concentration was slowly increased by 0.5 μM/week. After 6 months, metformin-resistant cell lines were obtained, termed 786-M-R, and maintained by culturing them in the presence of metformin.

### Cell proliferation assays

Cell growth was assessed using the Cell Counting Kit-8 (DOJINDO) assay. Caki-1, ACHN, A498, and 786-O cells were seeded onto 96-well tissue culture plates (4000 cells/well). Cells were cultured in medium with VPA (1 mM), metformin (10 mM) or a combination of these two. At the time point of 24 h, 48 h and 72 h, the medium was removed, and changed to 100 μl medium with 10% CCK8 reagent. Incubation time was 1 h. The absorbance at OD450 of each well was detected by a Thermo Scientific Multiskan GO (Thermo Fisher Scientific) machine. All CCK8 tests were performed in triplicate and repeated three times.

### Cell cycle and apoptotic analysis

Cell cycle analysis was performed using 786-O, and 786-M-R cells. Tumour cells were seeded in a 25 mm^2^ bottle and co-cultured with VPA, metformin or a combination of the two for 24 h and 48 h. The cells were digested and collected, then fixed with pre-cooled 70% ethanol at 4 °C overnight. The fixed cells were washed twice with PBS and stained with Muse Cell Cycle Reagent (Merck Millipore, Darmstadt, Germany). The stained cells were detected by Muse Cell Analyzer (Merck Millipore) to analyse the cell cycle distribution. The number of gated cells in the G0/G1, G2/M or S-phase is presented as the %. Cell apoptotic analysis was performed using 786-O, and 786-M-R cells. Tumour cells were seeded in a 25 mm^2^ bottle. After co-culture with VPA, metformin or a combination of the two for 48 h, the cells were digested and collected, then stained by 2uL Annexin V-FITC and 2uL Propidium iodide (BD Pharmingen, San Diego, CA, USA) every 100 μL of the cell suspension. After 15 min incubation at room temperature, the stained cells were detected by BD LSRFortessa to analyse the cell apoptotic distribution. All flow cytometry tests were repeated three times and representative results are shown.

### Immunofluorescence assays

RCC cells were fixed with 3% paraformaldehyde and permeabilized in 0.1% Triton X-100 (Solarbio Science & Technology Co., Ltd., Beijing, China). Then the cells were incubated with the appropriate primary and secondary antibodies, and the DNA was stained using DAPI (Solarbio). The immunostained cells were photographed with an inverted fluorescence microscope.

### Western blot analysis

RIPA Lysis and Extraction Buffer (Thermo Fisher Scientific) were used to extract the proteins from cells. Protease (Thermo Fisher Scientific) and phosphatase inhibitors (Roche, Basel, Switzerland) were added in the buffer to inhibit the activations of protease and phosphatases. Then the proteins were heated with 4× loading buffer at 95 °C, 10 min. After detecting the protein concentrations by the Micro BCA Protein Assay Kit (Thermo Fisher Scientific), we separated twenty micrograms of total protein lysate by SDS-PAGE and electro-transferred onto a polyvinyl difluoride membrane. The blots were blocked in Tris-buffered saline with 0.1% Tween 20 (TBST) and 5% milk or 5% BSA for 1 h at room temperature, then incubated with primary antibodies against pAMPK, p-AKT, total AKT, pSMAD3, SMAD3, SMAD4, aH3, H3 (Abcam) and GAPDH (Santa Cruz Biotechnology, Santa Cruz, CA, USA) overnight at 4 °C. Subsequently, the membranes were washed with TBST for three times and incubated with HRP-conjugated second antibodies (Santa Cruz) for 1 h at room temperature. The immune-reactive bands were detected by an Immobilon Western Chemiluminescent HRP Substrate kit (Merck Millipore). Pictures of the bands were recorded by Amersham Imager 600 (GE Healthcare, Little Chalfont, Buckinghamshire, UK). All the western blot experiments were repeated at least three times.

### Wound healing assays

We marked the wells of 6-well plates with straight black lines on the bottom, seeded the cells into the 6-well plates and cultured until cells reached confluence. Then cells were starved in medium with 0.1% FBS for 8 h. Three straight scratches were made by a 200-μl pipette tip across the black line in each well for the simulation of wounds. After gently washing off the loose cells with PBS, we added medium (0.1% FBS) with VPA (1 mM) and/or metformin (10 mM) or medium (0.1% FBS) alone into the wells and incubated for 18 h. Microphotographs were recorded immediately after the scratch (0 h) and at 18 h. The locations of images were aligned according to the black lines on the bottom. The wound healing assays were conducted in triplicate.

### Migration assays

Migration assays were performed by using a Boyden chamber containing 24-well transwell plates (Corning Inc. NY. USA) with 8-mm pores on the membrane. We trypsinized the cells, washed the cells twice with FBS free medium and then seeded approximately 7.5 × 10^4^ cells onto the upper chamber. As a chemoattractant, medium containing 10% FBS was added into the lower chamber and the plates were incubated at 37 °C in a 5% CO_2_ atmosphere for 24 h. The cells on the topside of the membrane were removed by scrubbing with cotton. After that, the membranes were fixed by pre-cooled 95% ethanol and stained by 0.1% crystal violet. Five random fields in each membrane were photographed to count the cell number. All experiments were repeated three times.

### Statistics

All experiments were performed at least in triplicate. The data were analysed with SPSS for Windows Statistics version 20 software (SPSS Inc. Chicago, IL). The 50% inhibiting concentration (IC50) values were calculated by linear regression analysis. Mauchly’s Test of Sphericity, ANOVA and MANOVA were used to analyse the CCK8 results. ANOVA and Paired T test were used to analyse the cell cycle tests, migration and wound healing assay results. Values of *P* < 0.05 were considered statistically significant.

## Results

### The combination of VPA and metformin

**Fig. 1:**
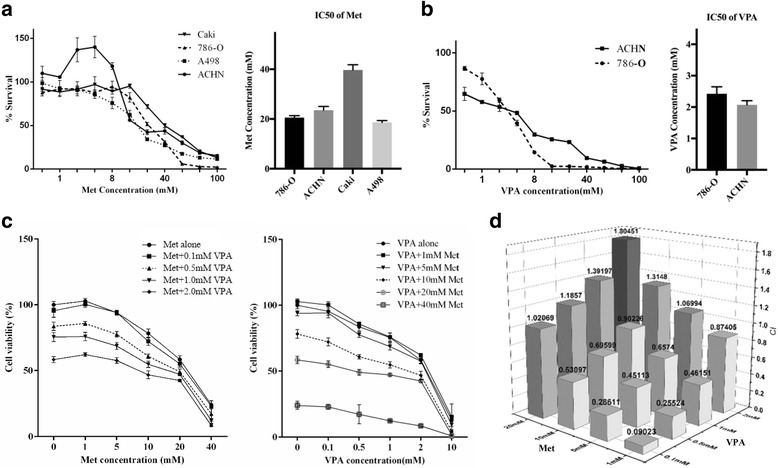
The effects of metformin and VPA on RCC cells proliferation. **a**, **b** RCC cells were incubated with increasing concentrations of metformin (Met) or VPA. Survival rates (%) were compared with the corresponding untreated cells. The IC50 values of metformin or VPA were calculated by SPSS software. **c** RCC cells were incubated with increasing concentrations of VPA and metformin combination for 48 h. Survival rates (%) were compared with the corresponding untreated cells. **d** The combination index (CI) of different combination groups with various concentrations of VPA and metformin

We first determined the IC50 values of metformin and VPA in RCC cell lines (Fig. 1a and b). For metformin: The IC50 of 786-O cell lines was 20.57 ± 0.77 mM, ACHN’s was 23.52 ± 1.52 mM, Caki-1’s was 39.73 ± 2.07 mM and A498’s was 18.63 ± 0.73 mM. The experimentally derived IC50 values of VPA were 2.424 ± 0.22 mM (786-O) and 2.073 ± 0.13 mM (ACHN). We used the median effect analysis, described before by Chou, TC, et, al [39], to evaluate the combination index (CI) of metformin (1, 5, 10 and 20 mM) in combination with VPA (0.1, 0.5, 1 and 2 mM) in 786-O (Fig. 1c and d). CI=CA/IC50A + CB/IC50B, CA and CB were the concentrations of drugs A and B. CI < 1 mean a synergy effect, and CI > 1 indicated an antagonism effect under the combination of two drugs. CI value of the group that VPA 1 mM in combination with metformin 10 mM was 0.90226. We chose this group of concentrations to make sure that at these concentrations drugs will have strong enough effects to observe as well as have a synergy effect on cells.

### VPA and metformin combination has a more pronounced inhibitory effect on RCC than metformin alone

**Fig. 2:**
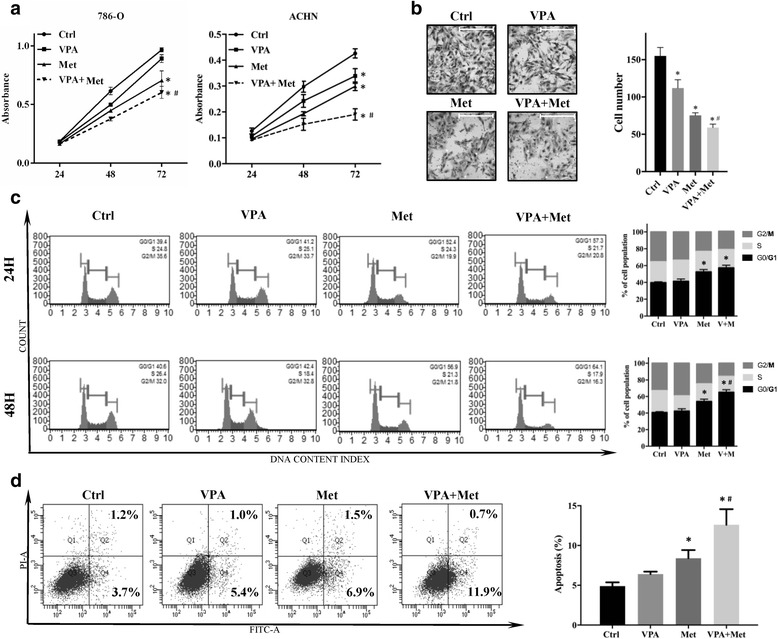
RCC proliferation, migration, cell cycle and apoptosis under VPA and metformin application (**a**) 786-O cells and ACHN cells were cultured with VPA 1 mM (VPA), metformin 10 mM (Met) or a combination (VPA + Met), and the cell viabilities were examined by CCK8 at 24, 48 and 72 h. *P < 0.05 vs control group, ^#^*P* < 0.05 vs metformin group. **b** The migration ability of 786-O cells was tested by transwell after treatment with VPA, Met or VPA + Met. Photos were taken at 200× magnification. Bar, 100 μm. The cell numbers of groups were counted. *P < 0.05 vs control group, ^#^*P* < 0.05 vs metformin group. **c** 786-O cells were treated with VPA, Met or VPA + Met for 24 h and 48 h then fixed by cold alcohol. The stained cells were detected by Muse Cell Analyzer to analyse the cell cycle distribution. Results were shown in bar graphs. *P < 0.05 vs control group, ^#^*P* < 0.05 vs metformin group. **d** 786-O cells were treated with VPA, Met or VPA + Met for 48 h. The rates of apoptosis were determined and analysed. *P < 0.05 vs control group, ^#^*P* < 0.05 vs metformin group

786-O and ACHN cells were cultured with VPA (1 mM), metformin (10 mM), or a combination, and the viability of cells was examined after 24 h, 48 h and 72 h. As shown in Fig. 2a, metformin alone remarkably inhibited the proliferation of 786-O and ACHN cells, VPA alone showed an un-significant but decreased proliferation activity in 786-O cells and a significant inhibition in proliferation activity of ACHN cells, however the combination of VPA and metformin inhibits proliferation much more than metformin alone. To detect the drug effect on tumour cell invasion, we performed a transwell assay. VPA (1 mM) or metformin (10 mM) alone can remarkably decreased the migration ability of 786-O cells, and the combination of VPA and metformin further enhanced this effect (Fig. 2b). In addition, we analysed the changes of the cell cycle in 786-o cells treated with VPA (1 mM) or metformin (10 mM) alone or combination for 24 h and 48 h. The flow cytometry analysis results revealed that metformin significantly increased the number of 786-O cells arrested in G0/G1 phases and VPA show no apparent effects on cell cycle. But in VPA and metformin combination group, the percentage of G0/G1 phases cells was much larger than metformin alone (Fig. 2c). The apoptotic analysis showed that, compared to control and metformin (10 mM) alone, VPA (1 mM) and metformin (10 mM) combination strongly increased the rates of apoptotic 786-O cells (Fig. 2d). These in vitro data suggested that VPA treatment alone has no distinct effects on RCC proliferation, cell cycle arrest and cell apoptosis, but shows inhibition on RCC migration. Metformin alone has antitumor activities which is consistent with previous laboratory studies, while the anti-tumour effects of metformin could be further enhanced by combination with VPA.

### VPA reverse metformin resistance in metformin-resistant RCC cells

**Fig. 3:**
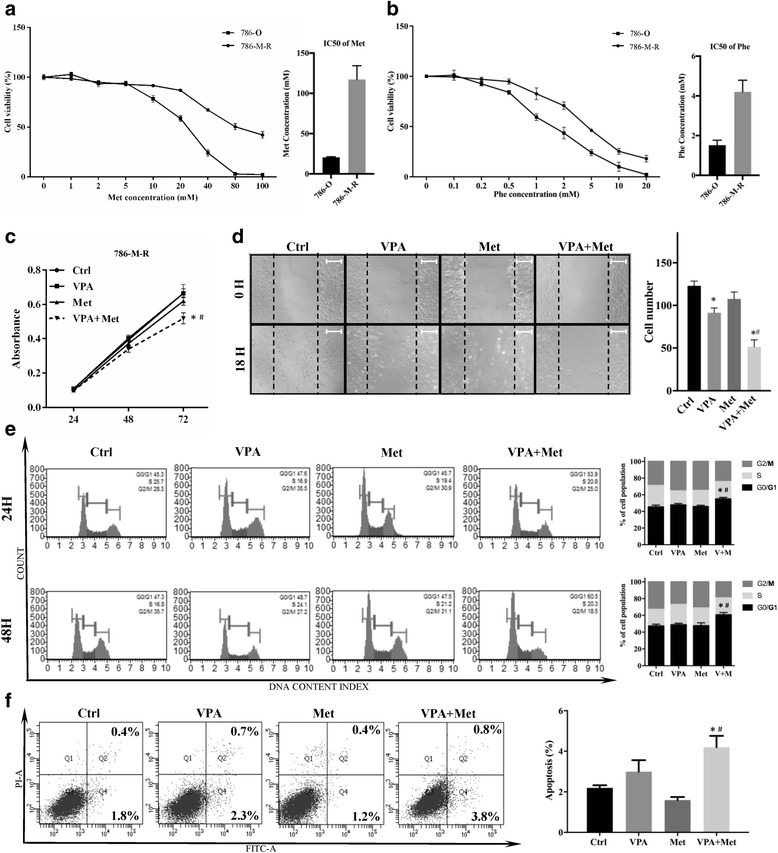
Reversing effect of VPA in 786-M-R cells. **a** The metformin resistant 786-O cells (786-M-R) were incubated with increasing concentrations of metformin for 48 h. Cell viability (%) was compared with the corresponding untreated cells. The IC50 values of metformin were calculated by SPSS software. **b** 786-M-R cells were incubated with increasing concentrations of phenformin for 48 h. Cell viability (%) was compared with the corresponding untreated cells. The IC50 values of phenformin were calculated by SPSS software. **c** 786-M-R cells were cultured with VPA 1 mM (VPA), metformin 10 mM (Met) or a combination (VPA + Met), and the cell viability was examined at 24, 48 and 72 h. ^#^*P* < 0.05 vs metformin group. **d** Confluent 786-M-R cells were starved for 18 h, scratched and grown in the presence of VPA, metformin or VPA + Met. Photos were taken at 200× magnification at the 0 h and 18 h. Bar, 50 μm. The cell numbers of groups were counted. *P < 0.05 vs control group, ^#^*P* < 0.05 vs metformin group. **e** 786-M-R cells were treated by VPA, Met or VPA + Met for 24 h and 48 h then fixed by cold alcohol. The PI stained cells were detected by BD LSRFortessa to analyse the cell cycle distribution. Results were shown in bar graphs. *P < 0.05 vs control group, ^#^*P* < 0.05 vs metformin group. **f** 786-M-R cells were treated with VPA, Met or VPA + Met for 48 h. The rates of apoptosis were determined and analysed. *P < 0.05 vs control group, ^#^*P* < 0.05 vs metformin group

The IC50 change is used to define drug resistance [40]. Our CCK8 results showed that, after 6 months application, 786-M-R cells exhibited a 5.7-fold higher IC50 than 786-O cells and after administration 40 mM metformin, the viability of 786-M-R cells was 67.22%, which was higher than 786-O cells (31.36%) (Fig. 3a). Figure 3b revealed that 786-M-R cells showed a cross resistant to phenformin, another biguanide [41]. The IC50 of phenformin in 786-M-R cells was 4.28 mM (1.51 mM in 786-O cells). In 786-M-R cells, metformin alone failed to inhibit cell proliferation, while VPA + Met significantly repressed 786-M-R cell proliferation (Fig. 3c). Through the wound healing assay, we found that 786-M-R cells treated with VPA + Met migrated much slower than treated with metformin alone, and VPA treatment impeded the migration of 786-M-R cells (Fig. 3d). Besides, metformin alone did not increase the number of G0/G1 786-M-R cells, but the combined treatment of VPA and metformin remarkably increase the number of cells arrested in G0/G1 phase compared to the control and Met groups (Fig. 3e). We also assessed the apoptosis and our data showed a significant decreased apoptosis was induced in 786-M-R cells by the drug combination (Fig. 3f). Clearly, combination with VPA can reverse the non-sensitivity of 786-M-R cells to metformin.

### The effects performed by VPA and metformin on pathways in RCC cells

**Fig. 4:**
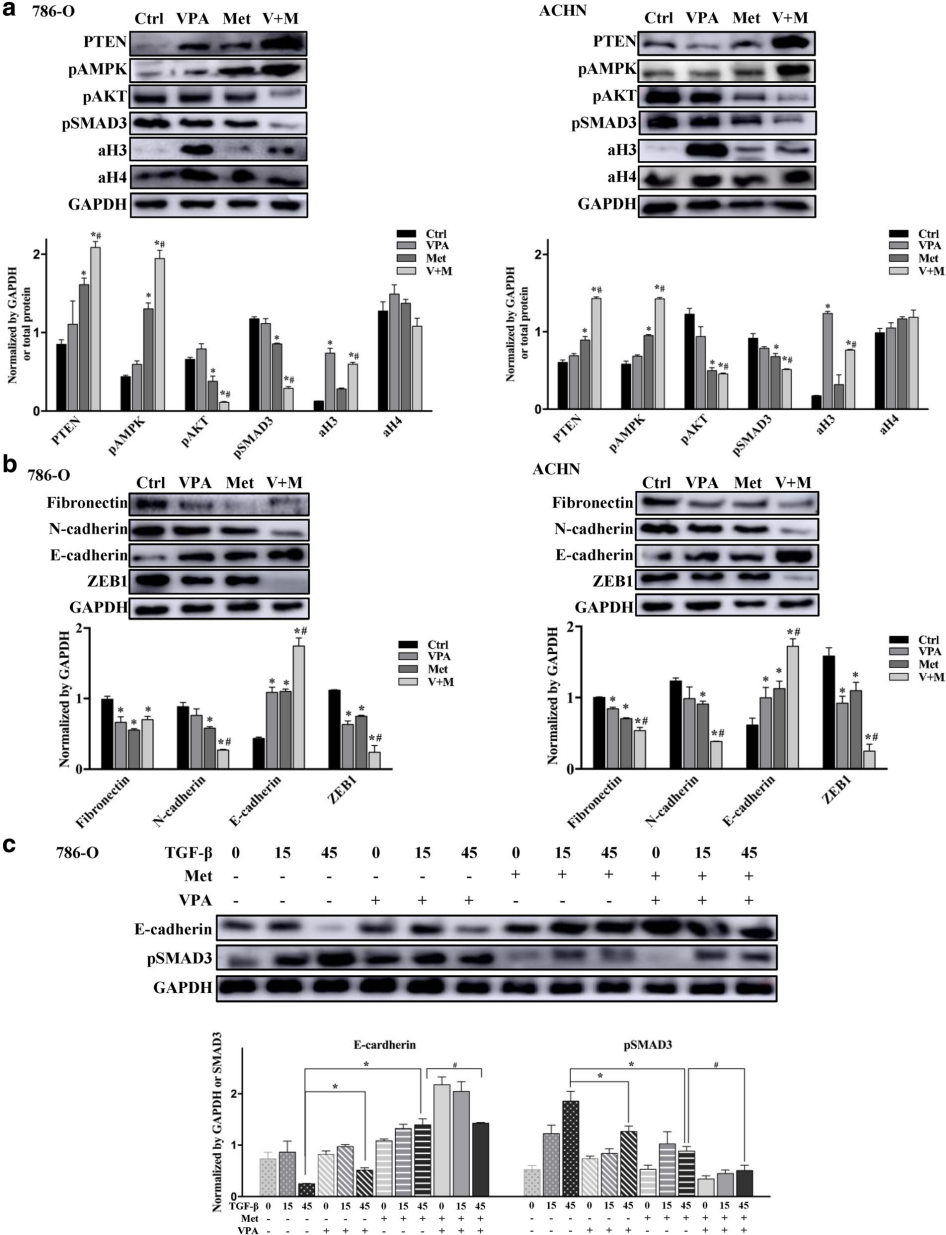
The effects of VPA and metformin in AKT and TGF-β induced EMT pathways in RCC cells**. a** 786-O cells and ACHN cells were cultured with VPA 1 mM (VPA), metformin 10 mM (Met) or a combination (V + M) for 48 h. The proteins were analysed by western blot and quantified by Image J. *P < 0.05 vs control group, ^#^*P* < 0.05 vs metformin group. **b** 786-O cells and ACHN cells were cultured with VPA, Met or a combination (V + M) for 48 h. The EMT markers and stem-like marker were analysed by western blot and quantified by Image J. *P < 0.05 vs control group, ^#^*P* < 0.05 vs metformin group. **c** After incubated with TGF-β (2 ng/ml) for 0, 15, 45 mins, pSMAD3 and E-cadherin in 786-O cells were analysed by western blot. Bands were quantified by Image J. *P < 0.05 vs control group, ^#^*P* < 0.05 vs metformin group

To identify the molecular mechanisms underlying the combination of VPA and metformin, we examined the effects of VPA and metformin on AMPK/AKT and TGF-β/SMAD3 signalling, which were reported to be the key pathways affected by metformin [42, 43]. As shown in Fig. 4a, in 786-O and ACHN cells, metformin alone remarkably increased the phosphorylation of AMPK and the expression of PTEN and decreased the phosphorylation of AKT and SMAD3; VPA alone significantly increased the level of aH3 and reduced the phosphorylation of SMAD3. Furthermore, comparing to metformin alone, the combination of VPA and metformin not only strongly enhanced the phosphorylation of AMPK and the expression of PTEN and inhibited the phosphorylation of AKT, but also decreased pSMAD3 and altered H3 acetylation.

In Fig. 4b, western blot analysis demonstrated that VPA treatment in RCC remarkably decreased the expression of fibronectin and ZEB1 and increased E-cadherin; metformin significantly decreased the expression of fibronectin, N-cadherin and ZEB1 and increased E-cadherin. As in combination of VPA and metformin, the effects of metformin performed on N-cadherin, fibronectin, ZEB1 and E-cadherin were strongly enhanced. These results confirmed that EMT was inhibited by VPA as well as metformin, and this inhibition was further enhanced by VPA and metformin combination. Then we analysed the two drugs’ roles in the process that using TGF-β to induce EMT. 786-O was incubated with TGF-β (2 ng/ml) for 0, 15, 45 mins then collected the total protein. Western blotting showed that the inhibition on TGF-β induced EMT process caused by VPA and metformin combination was much stronger than VPA and metformin alone, and was mainly rely on the decrease of pSMAD3 (Fig. 4c).

**Fig. 5:**
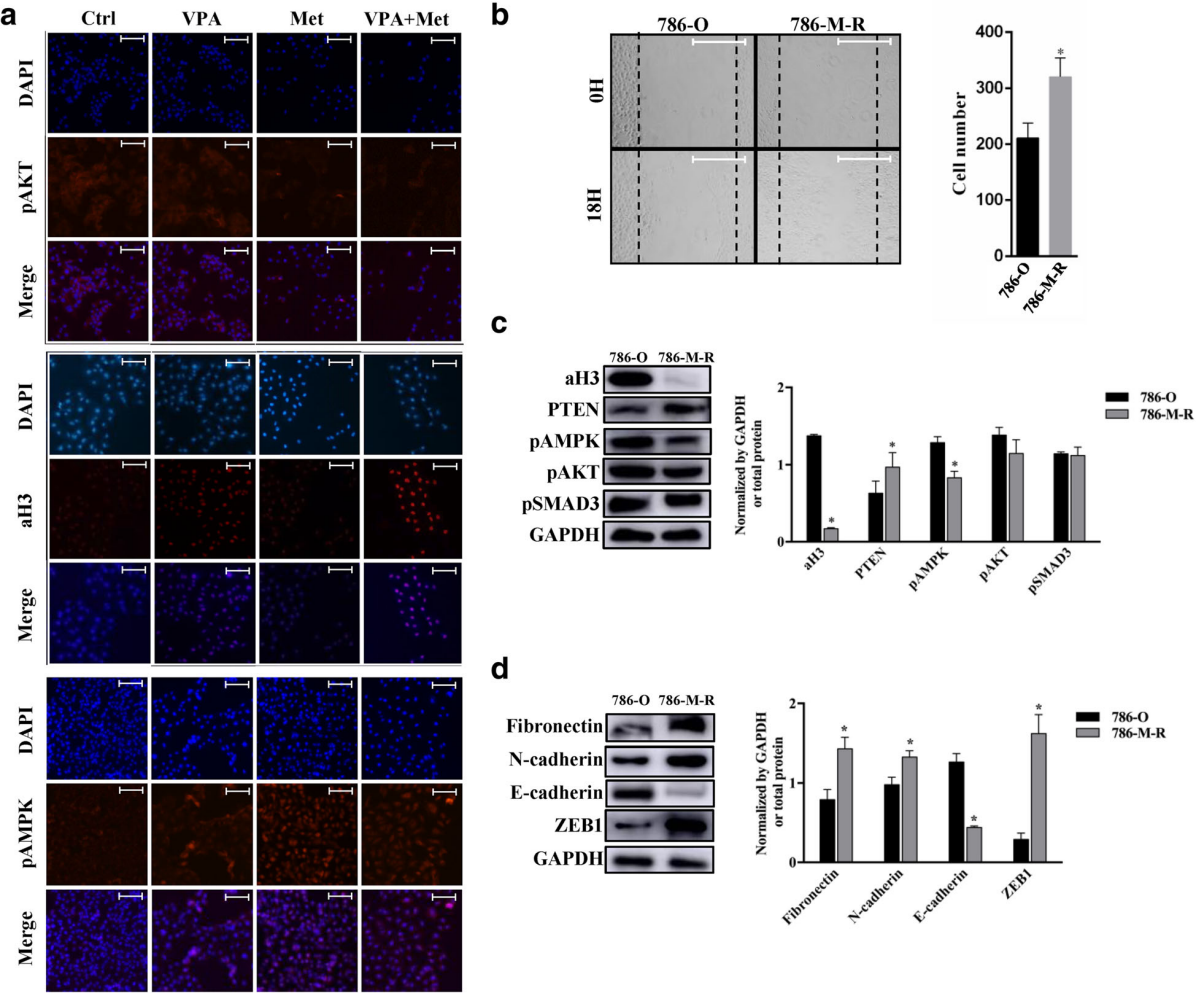
The difference between 786-O and 786-M-R cells in cell migration and proteins. **a** Graphs of Immunofluorescence. PAMPK, pAKT and aH3 were analysed by immunofluorescence in 786-O cells cultured with VPA 1 mM (VPA), metformin 10 mM (Met) or a combination (VPA + Met). (200× magnification; Bar, 50 μm). **b** 786-O and 786-M-R cells were starved for 12 h then performed wound healing assay, wound closure was calculated. *P < 0.05 vs 786-O group. (200× magnification; Bar, 100 μm). **c**, **d** Western blot analysis of cell signalling proteins and EMT makers in 786-O and 786-M-R cells. *P < 0.05 vs 786-O group

786-O cells were treated with VPA (1 mM), metformin (10 mM) or a combination for 48 h. Then we performed immunofluorescence analysis of aH3 expression and the levels of pAMPK and pAKT in different groups (exposure time: 6 s) (Fig. 5a). The pictures showed that metformin administration resulted in pAKT inhibition, and the inhibition was significantly enhanced by the VPA and metformin combination. Besides, the combination of VPA and metformin lead to a much stronger upregulation of pAMPK than the groups treated by VPA and metformin alone. The expression of aH3 were increased by VPA alone and the combination can remarkably elevate the increase of aH3. These pictures supported the results of western blot.

### The combinatorial effect of VPA with metformin in metformin-resistant 786-O cells

We first investigated the change in migration abilities and protein expressions in 786-M-R. Results of wound healing assay showed that 786-M-R cells were migrated faster than 786-O cells (Fig. 5b). The 786-M-R cells showed a decrease in levels of pAKT (not significant) and pAMPK and an increase in PTEN expression, however we observed that aH3 was dramatically reduced in 786-M-R compared to 786-O cells (Fig. 5c). In addition, sustained metformin exposure in 786-M-R cells induced the expression of EMT and stem-like makers. The expression of Fibronectin, N-cadherin and ZEB1 were elevated, whereas the epithelial marker E-cadherin was down regulated (Fig. 5d). These results suggested that EMT process altered in 786-M-R.

**Fig. 6:**
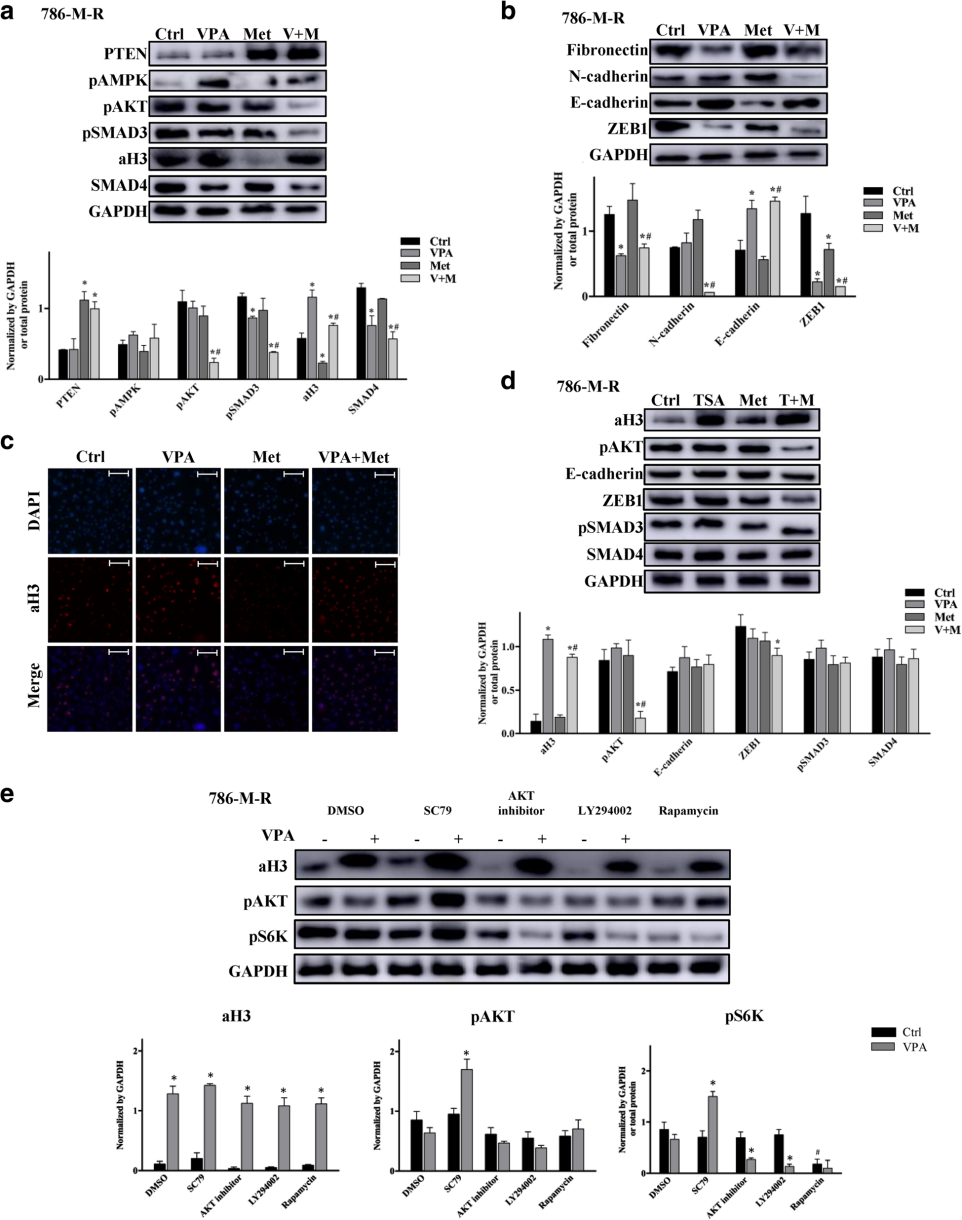
Effects of VPA, metformin or their combination on the pAKT pathways, EMT markers, the stem-like marker and histone 3 acylation in 786-M-R. **a**) 786-M-R cells were cultured with VPA 1 mM (VPA), metformin 10 mM (Met) or a combination (V + M) for 48 h. Western blot analysis of cell signalling proteins was performed. *P < 0.05 vs control, ^#^*P* < 0.05 vs metformin group. **b** 786-M-R cells were cultured with VPA 1 mM (VPA), metformin 10 mM (Met) or a combination (V + M) for 48 h. Western blot analysis of EMT markers and stem-like marker was performed. *P < 0.05 vs control, ^#^*P* < 0.05 vs metformin group. **c** Immunofluorescence analysis of aH3 expression in 786-M-R cells treated with VPA, metformin (Met) or their combination (VPA + Met). (200× magnification; Bar, 50 μm). **d** 786-M-R cells were cultured with thrichostatin A 200 nM (TSA), metformin 10 mM (Met) or a combination (T + M) for 48 h. Western blot analysis of aH3, pAKT, pSMAD3, SMAD4, E-cadherin and ZEB1 were performed. *P < 0.05 vs control, ^#^*P* < 0.05 vs metformin group. **e** The effects on histone 3 acetylation and pAKT in 786-M-R cells treated with various drugs that regulate AKT signalling. AKT inhibitor IV, AKT inhibitor; SC79, AKT activator; LY294002, PI3K inhibitor; Rapamycin, mTORC1 inhibitor; and DMSO as control. *P < 0.05 vs control, ^#^*P* < 0.05 vs DMSO group

The 786-M-R cells were treated by VPA 1 mM (VPA), metformin 10 mM (Met) or a combination (V + M) for 48 h. The expression levels and activity of the drugs’ target proteins and EMT and stem-like proteins were then detected by western blot. As shown in Fig. 6a, metformin’s effect on the levels of pAMPK, pAKT and pSMAD3 was not as remarkable as PTEN, but when combing with VPA, there showed a distinct reduced expression of pAKT and pSMAD3, suggesting the pAMPK, pAKT, pSMAD3 pathway were insensitive to metformin in 786-M-R cells. Besides, although previous results showed aH3 was loss in 786-M-R, but it still could be upregulated by VPA and VPA + Met. The immunofluorescence analysis of the aH3 expression in 786-M-R cells also proved that the VPA or VPA and metformin combination can upregulate the aH3 expression (Fig. 6c). In 786-M-R cells, metformin treatment had no significant effects on EMT proteins, however, the VPA and metformin combination counteracted this process and significantly decreased the Fibronectin, N-cadherin and ZEB1, and increased the E-cadherin (Fig. 6b). Besides, we tested the functions of metformin in combination with thrichostatin A (TSA) (200 nM), another HADCi. As shown in Fig. 6d, TSA and metformin combination could remarkably inhibit the level of pAKT but show no significant effects on EMT markers. We speculate that because VPA can reduce the expression of SMAD4, therefore down regulate the level of pSMAD3/SMAD4, while TSA show no influence on SMAD4. Next, we interrogated the potential role of AKT signalling pathways and the losing histone acetylation in 786-M-R cells. Cells were treated with AKT activator (SC79), AKT inhibitor (AKT inhibitor V, LY294002) and rapamycin with or without VPA. The adding of VPA can remarkably alter levels of aH3. SC79 significantly increase the phosphorylation of AKT and S6K in the presence of VPA, suggesting the sensitivity of AKT was increased by aH3 upregulation (Fig. 6e).

### Morphology changes in RCCs

**Fig. 7:**
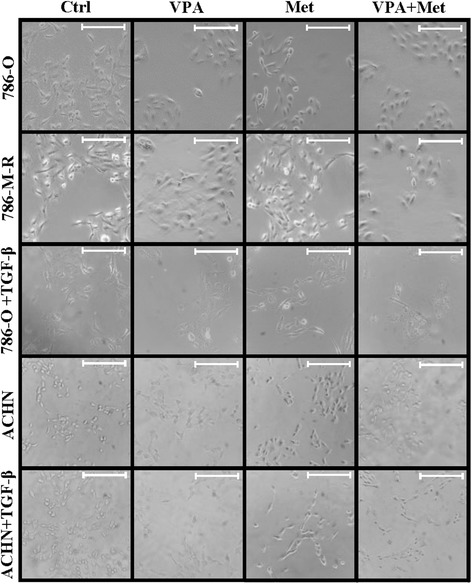
Morphology of RCCs under VPA, metformin or a combination treatment. 786-O, 786-M-R and ACHN cells were cultured with VPA 1 mM (VPA), metformin 10 mM (Met) or a combination (V + M) and with or without TGF-β (2 ng/ml) for 48 h. Cell morphology was observed by bright field phase microscopy at 200× magnification. Bar, 100 μm

We observed the morphology change in 786-O, 786-M-R and ACHN treated with and without TGF-β. As shown in Fig. 7, most of the 786-O and ACHN cells remained enlarged and had irregular nuclei, which suggested a higher invasive capacity. After VPA treatment, a higher number of flattened cells was observed. After metformin treatment, there appeared increasing numbers of well-stacked cells [44]. The combination of VPA and metformin can remarkably inhibit cell proliferation and the cell became round and short reflecting the low invasive capacity.

## Discussion

Although many cancers initially respond well to anti-tumour drugs, resistance occurs frequently. Different molecular mechanisms have been determined associate with resistance. It is urgently required that we need to discover more novel drugs and therapies against cancer. In recent years, many experimental studies revealed the remarkable antitumor activities of antidiabetic biguanide metformin in RCC [13–15], while the results of epidemiological studies and clinical trials are usually not consistent with the experimental studies [26–31]. The outcomes of metformin use in RCC patients are controversial in clinical trials, which makes metformin still far from acting as an antitumor drug alone. We assume that the long-term metformin treatment in clinical practice leads cancer cells resistant to metformin, resulting the effects of metformin on cancer risks reduced. In this study, we initially demonstrated that long-term treatment with metformin lead RCC became resistant to the drug. We built the metformin-resisted 786-O cell line (786-M-R) by cultured with stepwise concentrations of metformin increasing from 1.5 mM to 15 mM in 6 months. 786-M-R cells revealed a 5.7-fold higher IC50 than 786-O cells. Similarly, Scherbakov et al. induced resistance by long-term administration of metformin in breast cancer MCF-7 cells and found that the cell sensitivity to anti proliferative action of metformin were clearly inhibited [45]. Besides, we suspected the fact, that the concentrations of metformin used in experimental studies were much higher than therapeutic human plasma concentrations [46], should be responsible for limiting the extrapolation of these findings to clinical practice. But a previous study demonstrated that even at low physiological doses (0.3-1 mM) exposure to metformin dampened proliferation, colony formation and migration, and the anti-proliferative effects [47]. In fact, although the inhibitory effects in most of laboratory studies were observed at concentrations which are at least 10-fold higher than the peak plasma concentration attained with typical dosing in diabetics, there are emerging studies which show that even very low doses of metformin could have substantial anti-cancerous effects [48]. Therefore, the difference in concentrations may not explain the inconformity in clinical studies.

Among metformin targets, adenosine monophosphate-activated protein kinase (AMPK) should be mentioned which is responsible for large parts of metformin associated pathways. The phosphorylation of the activation loop threonine (Thr172 in human AMPKα1) is absolutely required to activate all known AMPK homologues. Metformin can activate AMPK with the presence of liver kinase B1 (LKB1), therefore link to the PI3K/AKT/mTOR pathway. RCC is a kind of cancer characterized by the over-activation of AKT/mTOR pathway and concomitantly reduced expression level of PTEN [49]. In RCC cell lines 786-O and ACHN, the application of metformin perform inhibition on cell proliferation and induce cell cycle rest and cell apoptosis, may be caused by the upregulated levels of PTEN and pAMPK, resulting in the inhibition of pAKT. The application of metformin on 786-M-R cells did not present inhibition on cell proliferation and changes in cell cycle and apoptosis, demonstrating the long-term metformin administration over 6 months made the cells nonresponsive to metformin’s growth inhibitory effects. The most obvious changes in signalling proteins of 786-M-R cells are the upregulation of PTEN and the reduction of pAMPK and aH3. In 786-O and 786-M-R cells, the expression levels of PTEN after treatment by metformin has the same trend, predicting the resistance may target on other proteins. Previous studies reported pAMPK over-expression is correlated with cancer resistance [50], while other studies suggested metformin can resensitize multidrug-resistant breast cancer through activated AMPK pathway [51]. In present study, metformin treatment can’t efficiently activate the AMPK in 786-M-R cells, suggesting the resistance to metformin is partly based on the low sensitivity of AMPK. We observed a slight down-regulation of pAKT in 786-M-R cells, while unlike in 786-O cells, the levels pAKT did not remarkably reduced after metformin treatment, suggesting pAKT could also be a base of metformin resistance. We believe the conclusion can be supported by a previous study that found elevated levels of pAKT in hepatic tumour cell were related to the resistance of metformin [52]. The decrease of pSMAD3 is coupled to cell growth arrest and re-differentiation in RCC [53]. The expression levels of pSMAD3 in 786-M-R cells were not significantly different with in 786-O cells. But after treatment with metformin, the pSMAD3 expression did not decrease in resistance cell and seemed lost sensitivity to metformin. Totally, the results allowed to assume the resistance to metformin in RCC are mainly based on the low sensitivity of pAMPK, pAKT and pSMAD3.

As a kind of HDACi, VPA alone can dramatically increase the levels of aH3 in RCC. Comparing with metformin application alone, VPA and metformin combination showed remarkably growth in the levels of aH3 and pAMPK, and inhibition on pAKT and pSMAD3. In 786-M-R cells, metformin in combination with VPA markedly suppressed the proliferation and migration capacity and induced cell cycle arrest and cell apoptosis. Besides, western blot showed significantly increase in the levels of aH3 (but not in the levels of pAMPK), and obviously decrease in the levels of pAKT and pSMAD3, suggesting VPA can counteract the development of resistance to metformin in 786-M-R cells by regulating pAKT and pSMAD3. Furthermore, we use pAKT activator and inhibitor to detect the regulation between aH3 and pAKT in 786-M-R cells and find that upregulation of aH3 can alter the sensitivity of AKT, letting pAKT efficiently upregulated by SC79. The results suggest that the inhibition and low sensitivity of pAKT in 786-M-R cells may be resulted by loss of aH3. A recent study reported the similar connection with aH3 and pAKT in VPA treated DU-145 cells [54], and others showed AKT decrease in lysine K18 acetylation of H3 and phosphorylates H3-threonine 45 [55, 56]. But the regulation mechanisms of aH3 and pAKT are still unclear.

Epithelial-to-Mesenchymal (EMT), a malignant cancer phenotype characterized by aggressive invasion and metastasis and mainly drove by transforming growth factor β (TGF- β), had been determined efficiently repressed by metformin [57]. The present study investigated the potential roles of metformin in inhibiting EMT of RCC. The results showed that EMT in RCC cell lines was impeded by metformin short-term treatment, while EMT progress became overactive in 786-M-R cells and failed to be arrested by metformin treatment. Drug resistance is clearly associated with the EMT state in several cancer chemotherapies [58–60]. The present study showed that metformin inhibited TGF-β induced EMT in RCC cells, and this inhibition was enhanced by VPA. Lan et al. suggested that VPA can inhibit EMT through the dual suppression of SMAD4, which is a significant intra-nuclear element of TGF-β induced EMT binding with pSMAD3 [61]. It is reasonable to suggest that metformin application alone failed to arrest EMT in 786-M-R cells, but the combination of VPA can reverse EMT by inhibiting pSMAD3/SMAD4 levels, resulting in decreased fibronectin and N-cadherin and the up-regulation of E-cadherin and ZEB1, which were suggested as key drivers of the EMT genetic program.

**Fig. 8:**
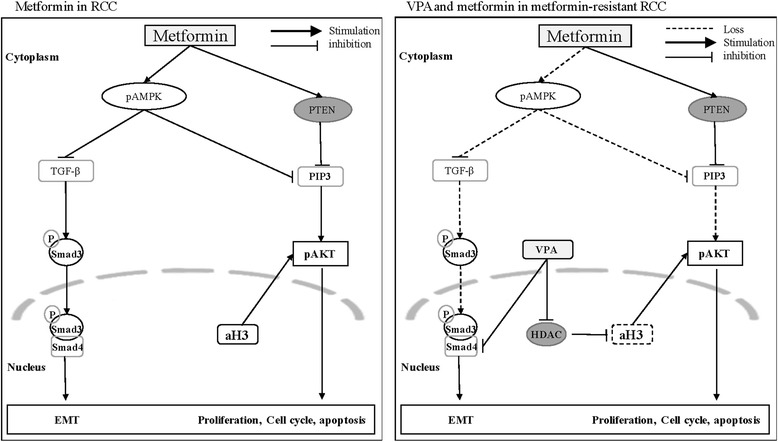
Tentative roles of aH3, pSMAD3, pAMPK and pAKT in RCC cells and metformin resistant RCC cells. Long-term application of metformin induces resistance in RCC and this resistance mainly based on the loss of sensitivities of AMPK/ AKT and TGF- β/SMAD3 pathways. However, the addition of VPA can counteract the resistance through increasing the sensitivities of pAKT by upregulation of aH3 and reversing the EMT process by inhibiting pSMAD3/SMAD4

Here, we provide a potential explanation to the inconformity of metformin’s antitumor activities between laboratory studies and epidemiologically analysis in RCCs. As pictured in Fig. 8, our data indicated that long-term application induces resistance to metformin in RCC and this resistance mainly based on the losing sensitivities of AMPK/ AKT and TGF- β/SMAD3 pathways. However, the addition of VPA can counteract the resistance caused by metformin long-tern application through upregulating of aH3, increasing the sensitivities of pAKT and reversing the EMT process. Finally, to support our findings, further clinical studies, animal studies and much deeper research on mechanism are needed and will be our further study focus.

## Conclusions

Long-term application of metformin results in distinct drug resistance. The resistance is mainly reflected in the loss of aH3 and the low sensitivities of AMPK/ AKT and TGF- β/SMAD3 pathways. Administration of VPA combined with metformin counteract the resistance of metformin in RCC through promoting the acetylation of H3 and reversing EMT.

## Abbreviations

AKT: Protein kinase B
AMPK: Adenosine 5′-monophosphate (AMP)-activated protein kinase
CCK8: Cell Counting Kit-8
EMT: Epithelial-to-mesenchymal transition
ERK: Extracellular regulated protein kinases
HDACi: Histone deacetylases inhibitor
IC50: The half maximal inhibitory concentration;
Met: Metformin
RCC: Renal cell carcinoma
VPA: Valproic acid
